# Kidney-on-a-Chip: Mechanical Stimulation and Sensor Integration

**DOI:** 10.3390/s22186889

**Published:** 2022-09-13

**Authors:** Dan Wang, Matthew Gust, Nicholas Ferrell

**Affiliations:** 1 Division of Nephrology, Department of Internal Medicine, The Ohio State University Wexner Medical Center, Columbus, OH 43210, USA; 2 Department of Statistics, College of Arts and Sciences, The Ohio State University, Columbus, OH 43210, USA

**Keywords:** kidney-on-a-chip, mechanical stimuli, microfluidic, glomerulus, proximal tubule, shear stress, substrate stiffness

## Abstract

Bioengineered in vitro models of the kidney offer unprecedented opportunities to better mimic the in vivo microenvironment. Kidney-on-a-chip technology reproduces 2D or 3D features which can replicate features of the tissue architecture, composition, and dynamic mechanical forces experienced by cells in vivo. Kidney cells are exposed to mechanical stimuli such as substrate stiffness, shear stress, compression, and stretch, which regulate multiple cellular functions. Incorporating mechanical stimuli in kidney-on-a-chip is critically important for recapitulating the physiological or pathological microenvironment. This review will explore approaches to applying mechanical stimuli to different cell types using kidney-on-a-chip models and how these systems are used to study kidney physiology, model disease, and screen for drug toxicity. We further discuss sensor integration into kidney-on-a-chip for monitoring cellular responses to mechanical or other pathological stimuli. We discuss the advantages, limitations, and challenges associated with incorporating mechanical stimuli in kidney-on-a-chip models for a variety of applications. Overall, this review aims to highlight the importance of mechanical stimuli and sensor integration in the design and implementation of kidney-on-a-chip devices.

## 1. Introduction

The kidney is a complex organ consisting of more than 20 cell types, including glomerular endothelial cells, podocytes, mesangial cells, and multiple segment-specific tubular epithelial cells. Collectively, these cells filter the blood, excrete toxins and metabolic waste, and reabsorb water and essential solutes from the glomerular filtrate, among other critical physiological functions [1,2]. The kidney microenvironment provides nutrients, a support scaffold, mechanical stimuli, and chemical signals that support normal physiological function [3,4]. Biochemical and biophysical cues can influence multiple cell functions including proliferation, differentiation, gene expression, signal transduction, migration, polarization, and cell survival [3,5,6]. Cells in the kidney are exposed to continuous mechanical stimuli from fluid flow, the surrounding extracellular matrix (ECM), or neighboring cells. Traditional in vitro cell culture models fail to replicate physiologically relevant mechanical stimuli that are important for regulating cell function [7].

Mechanical forces can affect kidney cell behavior that ultimately impacts organ function. Cells sense and translate mechanical inputs to activate cellular signaling through mechanotransduction [8]. Disease-mediated changes in mechanical signals, including altered glomerular and tubular fluid flow, pressure, and changes in the mechanical properties of the ECM, may all contribute to tissue damage and disease progression. Thus, incorporating mechanical stimulation into in vitro kidney models is important for understanding normal renal function in health, for understanding mechanisms of disease initiation and progression, and for developing therapeutic approaches to prevent or slow disease progression.

Kidney-on-a-chip technologies hold great potential to facilitate the design of kidney models that incorporate physiologically important aspects of the tissue microenvironment in vitro for studying basic mechanisms of kidney function and disease. In addition to studying kidney physiology and pathophysiology, kidney-on-a-chip devices have been widely utilized as platforms for nephrotoxicity screening. Kidney-on-a-chip has the potential to serve as miniaturized, low-cost bridges between overly simplistic in vitro cell culture models and expensive and complicated animal models. Additionally, fabrication techniques are amenable to rapid prototyping and iterative device design.

Devices can include single or multiple cell types inside a microchamber or microchannels with or without continuous flow and ECM to recapitulate the tissue microenvironment. Various kidney-on-chip devices have been developed to model the glomerulus [9,10,11,12] and the different segments of the tubule [13,14,15,16,17,18,19], two primary components of the nephron. Lithographic and 3D printing techniques have been used to build kidney-on-a-chip systems for multiple applications [20,21,22]. Proximal tubule cells, podocytes, endothelial cells, and mesangial cells from humans, mice, and other species have been grown in mono- or co-culture microfluidic devices to model renal physiology, pathophysiology, nephrotoxicity, and drug screening [9,16,23,24,25,26,27,28,29]. In addition, kidney-on-a-chip models incorporate mechanical stimulation in a variety of approaches by passively mediating substrate stiffness or geometry and actively applying forces. The integration of sensors in microfluidic devices enables real-time monitoring of cell function, growth rate, and cell monolayer integrity. Electrical, electrochemical, and optical sensors have been incorporated into various organ-on-a-chip systems [30,31,32,33]. However, except for measuring transepithelial electrical resistance (TEER), there are limited studies incorporating sensors into kidney-on-a-chip. These technologies may have the potential to further increase the applications and functionality of kidney-on-a-chip devices.

**Figure 1:**
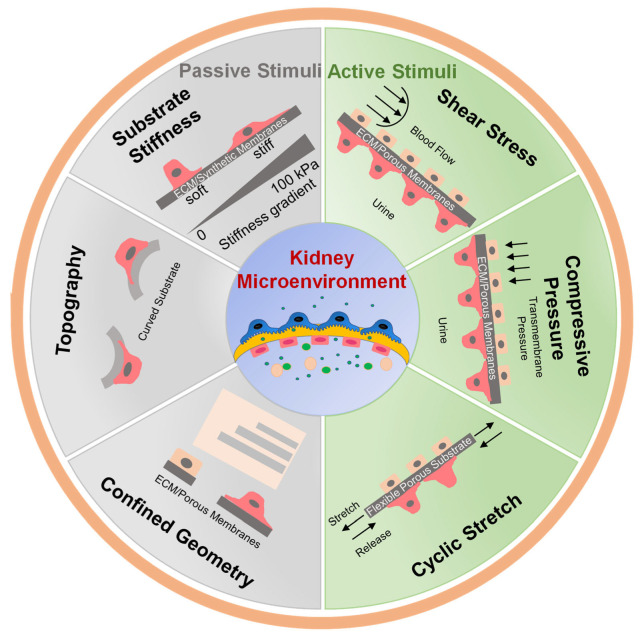
Kidney cell microenvironment and the mechanical stimuli applied to cells to mimic the physiological environment.

This review provides an overview of mechanical stimuli applied in kidney-on-a-chip devices and 3D in vitro models of the kidney. We provide an overview of passive biomechanical stimuli including incorporating synthetic, hybrid, and biological materials for modulating stiffness, surface topography, and confined geometry. We then discuss active stimuli, including fluid shear stress (FSS), compression, and cyclic stretch that are incorporated into kidney-on-a-chip models (Figure 1). We also review the integration of sensors in kidney-on-a-chip systems. Finally, challenges and future perspectives for kidney chips are discussed.

## 2. Importance of Mechanical Stimuli in Kidney

In vivo, kidney cells are subjected to complex and dynamic biomechanical stimuli that regulate cell behavior and function [3,34]. Mechanical stimulation has been incorporated in kidney-on-a-chip to recapitulate the biophysical microenvironment to study cell responses to different types of mechanical forces. Kidney cells are subjected to passive and active biomechanical stimuli in vivo [35]. Cells experience passive biomechanical forces through biophysical interactions at the cell-matrix interface. These include increases or decreases in ECM or basement membrane stiffness, geometrical confinement, changes in topography, or application of static stress [35,36]. Bioengineered, synthetic, and biological materials with or without topographic modifications have been integrated into kidney-on-a-chip to regulate cell-specific phenotypes and mimic physiological conditions [19,37].

Active mechanical stimuli include FSS, tensile stretch, and compression. The frequency, magnitude, and duration of active stimuli vary considerably between different cell types within the kidney. Glomerular endothelial cells are subject to apical FSS from blood flow that can be 30–50 dyn/cm^2^ [38,39,40], and shear stresses in the podocyte slit diaphragm have been estimated to reach 80 dyn/cm^2^ [41]. Shear stress on renal tubular epithelial cells is roughly an order of magnitude lower, with values of 2–4 dyn/cm^2^ in the proximal tubule [42]. Each individual cell type is highly sensitive to deviations in shear stress from the normal physiological set point [43,44,45]. A better understanding of how both passive and active biomechanical factors regulate kidney cell behavior and how variations in these forces induce pathological cell responses may provide new insight into normal physiological function and point to new pathways that contribute to the loss of kidney function in disease. Kidney-on-a-chip devices offer new opportunities to study these biophysical factors under well-controlled in vitro conditions that mimic specific aspects of the in vivo environment in health and disease.

## 3. Passive Mechanical Simulation

**Figure 2:**
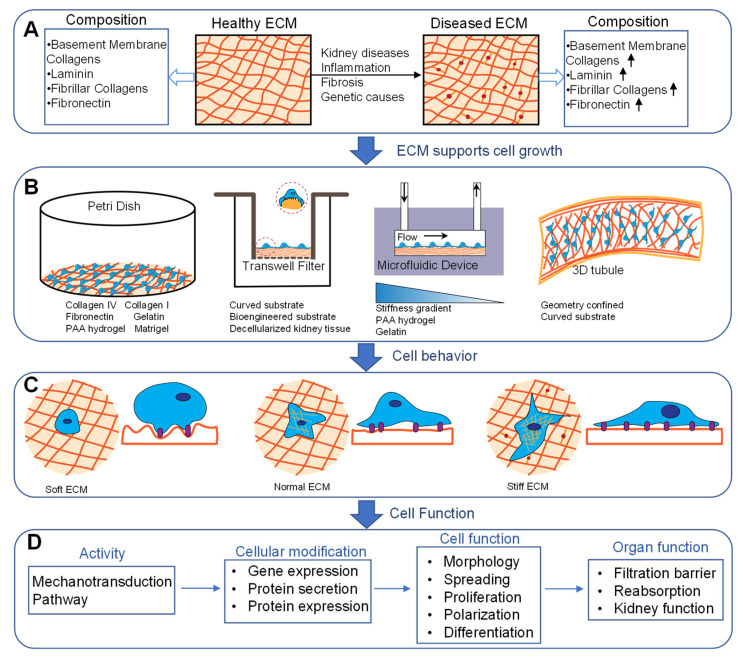
Passive mechanical stimuli applied to cells. (**A**) Healthy and diseased ECM. Healthy ECM with normal composition and elasticity. Disease-mediated changes modify the ECM composition and stiffness. Changes in ECM stiffness are mediated by enzymatic crosslinking, glycation, chemical reactions, or gene mutation. (**B**) In vitro models of passive mechanical stimulation include culture dishes coated with synthetic or ECM hydrogels with tunable stiffness, modified Transwell culture inserts, microfluidic devices, and 3D printed structures that allow for both passive and active mechanical stimuli. (**C**) ECM affects cell behavior when being cultured on soft, normal, or stiff substrate. (**D**) ECM active mechanotransduction pathways which alters the gene and protein expression. These modifications further affect cell function. Accumulation of these factors contributes to organ dysfunction.

Kidney cells are supported by cell-specific ECM networks or basement membranes that provide both structural support and signaling platforms that regulate cell function [46]. Glomerular endothelial cells and podocytes are supported by the glomerular basement membrane (GBM) which consists primarily of α345 collagen IV, laminin, nidogen, and heparin sulfate proteoglycans [47]. Tubular epithelial cell basement membranes are similar but consist of different collagen IV and laminin isoforms. Mesangial and interstitial cells are supported by their own compositionally unique ECM [48]. In disease, there are substantial changes to the composition, architecture, and biomechanics of these different matrices that may uniquely contribute to the loss of tissue function [49,50,51]. ECM mechanical properties, size, shape, topography, curvature, and geometry affect cell behavior and function. Approaches to control these parameters have been integrated into kidney chips to mimic physiological or pathological conditions (Figure 2).

### 3.1. Substrate Stiffness

The mechanical properties of the ECM regulate a host of cellular processes including proliferation, migration, survival, and differentiation [52,53]. Multiple biologically derived and synthetic materials have been used to study the effects of stiffness on kidney cell behavior. Biomaterials such as collagen, fibronectin, and gelatin hydrogels are used in both 2D and 3D cell culture models [54,55,56]. Polyacrylamide (PAA) gel, a synthetic material, is a widely used cell culture system due to the ability to simply and precisely control substrate mechanics over a wide range of stiffness [57]. The elasticity of a normal glomerulus is approximately 2.5 kPa measured by atomic force microscopy [58]. A decrease in the stiffness of the glomerulus has been observed in multiple kidney diseases [59,60]. As the tissue becomes fibrotic in chronic kidney disease and many other fibrotic diseases, ECM stiffness increases and drives further tissue damage [61]. Non-enzymatic glycation and the formation of advanced glycation end products (AGE) crosslink the kidney ECM and increase matrix stiffness in decellularized kidney ECM ex vivo [62,63]. These modifications may be relevant to diabetic kidney disease.

Multiple studies have evaluated the effects of stiffness on various kidney cell types including podocytes and tubular epithelial cells. Hydrolyzed PAA scaffolds with stiffness ranging between 0.6–44 kPa have been used to study podocyte differentiation and morphology [64]. The authors showed that substrate stiffness strongly influences podocyte morphology, elasticity, and podocin expression. To investigate cellular responses to chemical and mechanical cues, Garcia et al. designed a microfluidic device with varied chemistry and substrate stiffness [65]. In another study, a biomimetic gelatin-mTG cell culture platform was developed to evaluate podocyte protein expression during differentiation [66]. Podocyte-specific markers, such as WT-1, neph1, nephrin, and podocin gene expression were upregulated on substrates with intermediate stiffness (2–5 kPa) that is similar to renal tissue but with lower expression levels on stiffer or softer substrates. PAA gels with a stiffness gradient were fabricated by slide-mask photopolymerization [65]. MDCK cells cultured in the device showed a relationship between cell scattering and stiffness when stimulated with a gradient of hepatocyte growth factor (HGF). Kidney tubular epithelial cells have been grown on both synthetic and natural ECM substrates with wide-ranging stiffness to evaluate the effects of matrix mechanics on differentiation, proliferation, survival, and spreading [67,68,69,70]. Multiple studies have shown that epithelial cell differentiation or epithelial to mesenchymal transition (EMT) in response to pro-fibrotic growth factor is influenced by substrate stiffness. Kidney epithelial cells on soft surfaces were resistant to growth factor-induced de-differentiation [67,69] but were more prone to cell death [69].

### 3.2. Surface Topography and ECM Composition

Cells cultured in vitro face the limitation of loss of differentiated function and reduced expression of cell-specific proteins [71,72]. Engineered biomimetic substrates could serve as scaffolds to modulate cell morphology and gene expression, and preserve differentiated function in cultured cells. Proximal tubule cells and podocytes adhere to a naturally curved basement membrane. Substrate curvature has been found to affect renal epithelial cell behavior [73]. A kidney chip with curved surfaces was fabricated using a replica of a polydimethylsiloxane (PDMS) master, 3D printing, and PAA gels to mimic the physiological tubule environment. Renal epithelial cells cultured on convex and concave surfaces exhibit different morphology, alignment, and polarization as compared with flat surfaces. Korolj et al. developed a cell culture model of the glomerulus by incorporating an engineered porous PDMS membrane with a Transwell filter. The membrane was formed by replicating a PDMS mold from glass beads (<100 μm) embedded in a SU8 (50 μm thick)-coated silicon wafer. Curved surfaces with porous structures provided a biomimetic surface for podocyte culture. This bioengineered platform showed that topographical modification promotes podocyte differentiation and upregulated nephrin gene expression [74].

Recently, efforts have focused on incorporating the natural properties of the ECM or basement membrane composition and architecture into organ-on-a-chip and 3D culture models [75]. We developed a model of the glomerular filtration barrier using decellularized GBM to evaluate the effects of basement membrane damage on molecular permeability [76]. We showed that both the GBM and podocytes contribute to the diffusive permeability of the system, and hypochlorous acid-mediated damage to the GBM increases molecular permeability. Homan et al. created proximal tubules on a chip by depositing a thin layer of gelatin-fibrin hydrogel using 3D printing techniques [15]. This allowed for the precisely controlled size and composition of the tubule chip with an elastic modulus of the ECM (~3.5 kPa), similar to normal kidney tissue stiffness, thus recreating the biophysical properties, architecture, and composition of the healthy kidney while also perfusing the system. The same group further incorporated vascularization to evaluate tubule transport function [77]. Such devices that incorporate multiple physiologically important factors may be particularly useful tissue models.

### 3.3. Confined Geometry

Adherent cells attach and spread on the ECM, and controlling cell spreading using micropatterned ECM has significant effects on cell behavior [78,79]. A loss of normal epithelial morphology is associated with kidney disease. Bosch-Fortea et al. developed a micropatterned array to evaluate epithelial cell morphogenesis and drug toxicity [80]. Several tubule epithelial cell types were grown on different ECM micropattern shapes to control lumen formation with or without drug exposure. The model provides a method for fine-tuning the microenvironment for modeling epithelial morphogenesis. Cells in confined tubule-like geometries were more sensitive to nephrotoxicity agents such as gentamicin. Confined geometry has also been shown to regulate the collective cell migration of kidney tubule cells. MDCK cells that migrated into circular lumens of varying diameters showed differences in migratory behavior and alteration in cytoskeletal architecture [81]. Cells in smaller diameter tubes were more aligned and had slower migratory speeds than cells in larger channels.

## 4. Active Mechanical Simulation

The importance of active mechanical stimuli in models of kidney-on-a-chip is highlighted by numerous studies. Generally, these devices employ photolithography, soft lithography, or 3D printing to mold microchannels. Cells cultured on the surface of single or multiple microfluidic channels are perfused with a rotary or syringe pump to apply FSS to the apical cell surface. Other devices have been fabricated from hollow fibers with curved topography along with shear stress to simulate the physiological environment of the proximal tubule [82,83]. These bioreactor devices provide the additional advantage of supplying large surface areas with continuously applied shear stress. The appeal of 3D printing technology is highlighted by the capability of generating 3D microstructures using synthetic biomaterials. Given the complexity of the kidney, devices that combine mechanical stimulation with additional chemical and/or electrical stimuli may provide a more biomimetic microenvironment.

### 4.1. Fluid Shear Stress

**Figure 3:**
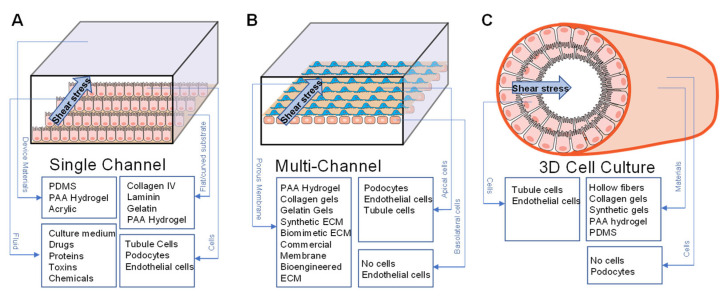
Shear stress applied to kidney-on-a-chip. Schematic drawing of the design for single-channel, multi-channel, and 3D cell culture devices. (**A**) A single channel is coated with collagen or laminin ECM, gelatin, or PAA hydrogel for cell attachment. Renal tubule cells, podocytes, or endothelial cells cultured on a thin layer of ECM. Cells are supplied with culture media and reagents for drug screening, toxicity evaluation, or interrogation of different cell functions. (**B**) Multi-channel device is composed of apical and basal channels with continuous media supply. Shear stress can be applied to each channel. Cell monolayer or co-cultured cells are supported by a bioengineered, synthetic, or commercially available porous membrane. (**C**) 3D cell culture device is composed of hollow fibers, 3D printed gels, or PDMS.

Blood flow in the kidney microvasculature or filtrate in the tubules creates a fluid force at the cell surface parallel to the direction of flow commonly referred to as fluid shear stress (FSS). FSS has been widely applied for physiological and pathological studies of the glomerulus, primary tubule, distal tubule, and collecting duct [84,85,86,87,88]. Multiple kidney cell types, including glomerular endothelial cells, podocytes, proximal tubules, and distal tubule cells, are all highly sensitive to FSS. In a rectangular microfluidic channel with laminar Newtonian fluids under steady conditions, FSS can be calculated by the equation: *τ =* 6 *μQ/bh*^2^, where *τ* is FSS, *μ* is the medium viscosity, *Q* is the flow rate, *b* is the width of the channel, and *h* is the height of the channel. Evaluating fluid forces due to blood in the glomerulus requires more complex modeling. Different techniques have been used to evaluate fluid forces in the glomerular capillary including FSS on the glomerular endothelium [38,39,40]. Shear stress can be applied to single channels, multiple channels consisting of an apical and basal chamber, and the 3D tube-like kidney-on-a-chip (Figure 3). The figure shows an enlarged channel to demonstrate the main components of the device. Most devices are fabricated with PDMS or acrylic by lithography techniques. Shear stress was applied to the device by connecting the inlet with a syringe pump or peristaltic pump. Cell culture media with reagents were perfused into the device. To model the filtration barrier, podocytes and endothelial cells were co-cultured and separated by a porous membrane in a kidney-on-a-chip with a flat or a 3D curved surface. The basic materials for fabricating the device and cell types for cell monolayer or co-cultured models are listed in Figure 3.

#### 4.1.1. FSS in Modeling Normal Physiology

The glomerulus is the filtering unit of the nephron and consists of fenestrated endothelial cells that line the capillary, the GBM, and podocytes on the filtrate side of the filtration barrier [89]. Both glomerular endothelial cells and podocytes are sensitive to FSS in vitro. Friedrich et al. cultured mouse podocytes in a commercial microfluidic device. Shear stress above 0.25 dyn/cm^2^ resulted in podocyte loss, cytoskeletal reorganization, and the activation of specific tyrosine kinases [43]. Huang et al. further showed that high shear stress-induced apoptosis via a c-Src and mTOR-mediated pathway [90]. FSS has also been shown to influence the differentiation of podocytes cultured in a microfluidic device [91]. Podocytes culture in 0.5–2 dyn/cm^2^ FSS, with or without retinoic acid, showed increased expression of podocyte-specific markers including synaptopodin, podocin, and WT-1 at the gene and/or protein level. This shows that mechanical stimulation with FSS effectively influences podocyte differentiation. As compared to static culture, podocyte-specific markers were significantly increased under FSS. Studies have further shown that shear stress regulates prostaglandin E2 and proteoglycan signaling in podocytes [92,93]. Glomerular endothelial cells have also been studied in the presence of FSS [87]. Glomerular endothelial cells cultured under long-term shear stress showed reduced NF-κB activation and PDGF-B expression [94], as well as increased KLF2 expression [95].

Significant effort has focused on modeling the proximal tubule due to its important role in water and salt reabsorption and the susceptibility of the proximal tubule to drug and toxin-induced injury [96,97]. The tubule consists of a single layer of polarized epithelial cells that transport water, glucose, proteins, amino acids, and other solutes including both anionic and cationic drugs. Solutes are reabsorbed by pumps, channels, and receptors present in the basolateral (interstitial side) and apical (tubular lumen side) membrane [98]. The apical surface of the proximal tubule is exposed to continuous flow. Tubular FSS depends on tubular fluid flow rate, viscosity, and tubule diameter. Shear stress is reduced in the distal nephron as water is reabsorbed and flow decreases [42,45].

Several studies have shown that proximal tubule cells are highly responsive to FSS. Duan et al. quantitatively evaluated changes in the actin cytoskeleton and cell–cell junctions in response to shear stress. Proximal tubule cells were exposed to FSS at 1 dyn/cm^2^ for 5 h. Their results showed reinforcement of peripheral actin bands and a tighter spatial distribution of ZO-1 and E-cadherin at cell junctions [99]. The same group also showed that FSS affects the localization and expression of apical and basolateral transporters in mouse proximal tubule cells [44]. Cells cultured in a parallel-plate flow chamber were exposed to 0.2 dyn/cm^2^ FSS for 3 h. FSS increased apical localization in NHE3, upregulated Na/K-ATPase expression and translocation, and induced V-ATPase trafficking. Several other studies have also observed changes in actin cytoskeletal architecture in response to FSS [23,100]. Several studies have also shown that FSS increases apical protein endocytosis in proximal tubule cells [26,101,102,103]. Proximal tubules are able to capture and process plasma proteins that traverse the glomerular filtration barrier. Under normal conditions, little protein filters into the tubule. In CKD, the filtration barrier is compromised and significant protein leaks into the filtrate. Weisz et al. have performed mechanistic studies to show that FSS regulates apical endocytic activity through cilia and mTOR-mediated pathways [101,102].

There are relatively few distal tubule kidney-on-a-chip devices compared to proximal tubule models. Baudoin et al. developed a microfluidic distal tubule in vitro model by culturing MDCK cells in a PDMS microchip with a flow rate of 10 μL/min [14]. At a high flow rate (50 μL/min), with 24 h of perfusion, the viability of the cells was reduced by 90%, indicating that high FSS induces cell death. Glucose consumption also increased at a higher flow rate. Another study reported a multi-layer microfluidic device fabricated using PDMS molds to create a static and a flow chamber [17]. Then, a porous membrane was embedded in the two chambers and bonded together. Primary rat inner medullary collecting duct (IMCD) cells were isolated and cultured in the microfluidic device. The cells were exposed to 1 dyn/cm^2^ of shear stress for 5 h after culturing on the membrane for 3 days. The cells expressed specific markers: AQP2 localized at the apical side and the Na-K-pump localized at the basolateral membrane, which was not observed in the cells cultured on glass. In addition to 2D models, a 3D kidney cortical collecting duct model was developed by Rein et al. using a pin-pullout technique [104]. The 3D channel was perfused with an ECM hydrogel, then mmpk cortical collecting duct cells were cultured in the device for 7 days under FSS (0.1 dyn/cm^2^). The model exhibited tight barrier function after diffusing FITC-dextran for 1 h. The cells showed polarization and transmembrane receptor expression.

Most microfluidic devices apply FSS using an external syringe pump or peristaltic pump. Other pumpless techniques have been used to apply FSS to kidney cells. Kimura et al. demonstrated a pumpless microfluidic device for culturing ureteric bud cells from mouse embryonic kidneys [105]. The microfluidic device comprised a medium tank and a microfluid chamber with cell culture and serpentine resistance channels. After the cells were subjected to FSS in the range of 0.4–0.6 dyn/cm^2^ for 48 h, the expression of tip cell marker genes was upregulated, but stalk cell marker genes were downregulated. The method provides a solution for culturing several plates under shear stress. Orbital shear stress has also been used to apply shear forces to kidney epithelial cells [101,106]. This was performed using an orbital shaker to apply fluid shear. While the shear stress is less uniform when applied with an orbital shaker, this approach is amenable to multiple culture plates and is less prone to issues such as bubbles that can be problematic in microfluidic systems.

#### 4.1.2. FSS in Modeling Disease

Microfluidic devices precisely control the cell microenvironment and provide more reliable drug toxicity screening and modeling of pathological conditions such as renal fibrosis and proteinuria. One of the primary applications of kidney-on-a-chip devices has been for screening drug nephrotoxicity. Proximal tubule toxicity is a primary off-target effect of many drugs such as chemotherapeutics and antibiotics. Kidney-on-a-chip devices for drug screening and nephrotoxicity have been reviewed elsewhere [24,107,108]. This discussion will only focus on the role of mechanical forces in regulating the uptake of nephrotoxic drugs. Several kidney-on-a-chip models of drug toxicity have shown the upregulation of drug transporters and/or increased drug uptake in tubular epithelial cells exposed to shear stress [109,110]. Yin et al. developed a co-culture microfluidic kidney chip with a temperature sensor and drug concentration gradient generator for drug screening and nephrotoxicity assessment [111]. Renal proximal tubule epithelial cells (RPTECs) and peritubular capillary endothelial cells (PCECs) were co-cultured in the device with a flow rate range of 10–100 μL/min. The concentration gradient chip was designed to obtain five different concentrations of drugs. Cisplatin, gentamycin (GM), and cyclosporin A (CsA) were injected into the chamber, and differences in cell viability were observed under static versus flow conditions. The mechanisms that regulate this process are not completely understood but may be cilia-mediated or cilia-independent [112]. Importantly, these devices may better recapitulate drug pharmacokinetics as compared to traditional cell culture systems or even animal models and therefore provide the potential for improving drug screening or developing strategies to minimize drug nephrotoxicity.

High blood pressure is one of the most common causes of chronic kidney disease. Hypertension may cause glomerular dysfunction by increasing filtration pressure. Zhou et al. developed a functional microfluidic chip to mimic hypertensive nephropathy [28]. The device consists of three layers: upper and lower PDMS chambers and a commercial porous polycarbonate membrane separating the two chambers. Glomerular endothelial cells and mouse podocytes were co-cultured in the device. To simulate hypertension, shear stresses of 0.001–0.003 dyn/cm^2^ were applied to the endothelial chamber. The results showed that with the FSS at 0.001 dyn/cm^2^, the barrier was restrictive to BSA and IgG transport, but permeability increased at high flow rates. The loss of selectivity was accompanied by a redistribution of F-actin, reduced CD-31 expression, and increased vWF expression in endothelial cells. Podocytes also showed cytoskeletal changes and reduced synaptopodin, nephrin, and podocin expression. These data suggest that high FSS and increased stretch increase glomerular permeability by damaging both endothelial cells and podocytes.

FSS was applied within the microfluidic device to model fibrosis in the renal tubule [13]. The device was fabricated using a rapid prototyping technique that consisted of two layers with 12 microchannels. Immortalized human renal proximal tubular cells (HK-2) were grown inside the culture chamber. The flow rate was set to 0.6 μL/mL to mimic physiological shear stress. Media containing TGF-β1, HHS, or C3a with different concentrations were used to simulate pathological conditions. The results show that the TGF-β1 caused morphology changes in HK-2 cells depending on the concentrations and culturing time.

#### 4.1.3. FSS in Modeling Barrier Function

The glomerular filter provides a size, charge, and shape-selective barrier that filters the blood by allowing small molecules and water to cross the barrier while retaining large proteins, such as albumin (3.5 nm hydrodynamic radius), in the plasma [113]. Podocytes wrap around the glomerular capillary, creating interdigitating foot processes that form slit diagrams. Podocytes are highly specialized terminally differentiated cells with minimal regenerative capacity after injury. Damage to any layer of the filtration barrier can result in the loss of glomerular selectivity and proteinuria that can progress to kidney failure [114,115]. In vitro models of the glomerulus have been developed using Transwell membranes to evaluate cell permeability [116,117]. Static models have the advantages of simple design and operation but cannot recapitulate the in vivo microenvironment with continuous flow and close contact between podocytes and glomerular endothelial cells. A study reported a glomerulus on a chip with co-cultured human podocytes and human glomerular endothelial cells with an artificial membrane [10]. This work demonstrated the device can maintain cell phenotype and function for at least one month. The cells were exposed to shear stress of 0.0117 Pa (0.117 dyn/cm^2^). The permselectivity of the filtration barrier was evaluated based on FITC-albumin transport across the filtration barrier. They used different combinations of cells and showed that the highest selectivity was achieved when podocytes and glomerular endothelial cells were co-cultured. They also demonstrated increased permeability when cells were treated with the puromycin aminonucleoside (PAN) or with serum from patients with membranous nephropathy. Xie et al. developed a glomerulus-mimicking knot with microscale hollow fibers with complex concave and convex topography to mimic the glomerulus in vitro [118]. Podocytes were cultured on the surface of the 3D knot while endothelial cells were cultured inside the hollow fiber to construct a filtration barrier. The FSS on the lumen side ranged from 0.3–0.9 Pa. The molecular permeability across the filtration barrier with or without cells was evaluated using Ficoll, bovine serum albumin (BSA), and inulin. The results showed a significant reduction in molecular transport across the filtration barrier with cultured cells as compared to cell-free.

### 4.2. Compressive Pressure and Cyclic Stretch

The pressure drop across the glomerular filter creates both compression and stretch in the capillary wall. Studies have shown that hypertension increases the pressure gradient to enhance mechanical stress on podocytes, resulting in podocyte loss [119]. As increased transmembrane pressure across the filtration barrier may cause kidney damage, Chen et al. designed a microfluidic device to study podocyte permeability in vitro [120]. Two acrylic chambers were separated by a collagen-coated anodic aluminum oxide membrane with nanoscale pores. The podocytes were cultured on the membrane until confluency. The pressure drop between the chambers was used to simulate the pressure drop between the glomerular capillary and Bowman’s space. The results showed that permeability increased with increasing pressure in the absence of any stretch. Increased net pressure downregulated the expression of synaptopodin and reorganized the actin cytoskeleton. This suggests that pressure is an important mechanical stimulus in the physiological or pathological glomerulus.

Most kidney-on-a-chip devices are designed as a single channel with applied shear stress that lacks the systematic analysis of cell functions. As the glomerulus is exposed to pulsatile blood flow, cyclic stress and shear stress need to be considered in recapitulating the human physiological response. Musah et al. developed a glomerulus-on-a-chip using stem-cell-derived podocytes to model the glomerular capillary wall. This system incorporated both FSS and cyclic stretch and showed synergistic effects of flow and strain on device function [11]. A porous PDMS membrane was sandwiched between two parallel microchannels in the multifunctional microfluidic device. Two hollow chambers were designed to apply a dynamic mechanical stretch. Podocytes were differentiated in 0.0007 and 0.017 dyn/cm^2^ FSS in the top and bottom chambers, respectively. A 10% cyclic strain (1 Hz) with shear stress exhibited a significant increase in podocyte-specific marker expression and increased VEGF-A secretion with flow and an additional increase when both flow and stretch were applied. This suggests that FSS and cyclic stretch have compounding beneficial effects on cell differentiation. They further showed that the permeability of the barrier increased with exposure to the cancer drug adriamycin.

## 5. Sensor Integration in Kidney-on-a-Chip

**Figure 4:**
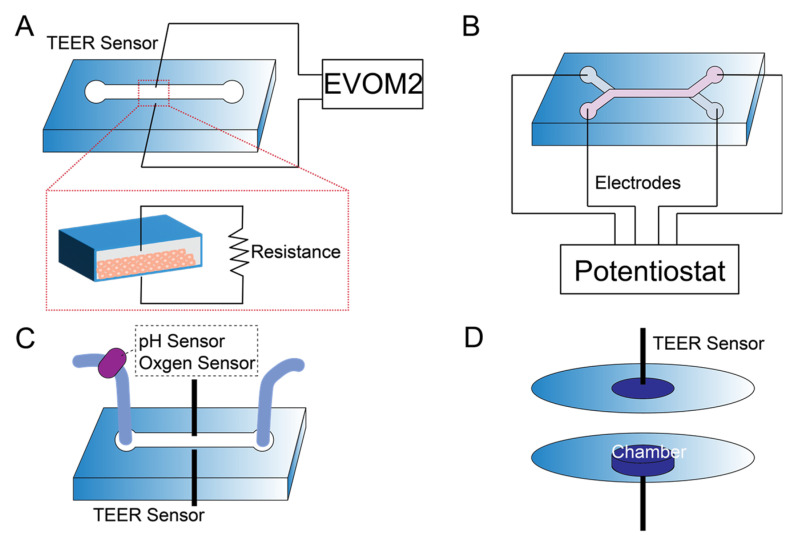
Sensor integration into kidney-on-a-chip with schematics of TEER measurement using (**A**) EVOM2 instrument and (**B**) four terminal sensing techniques using a potentiostat. TEER sensor measures the resistance across the cell layer. (**C**) Multiple sensors, such as pH and oxygen sensors, are integrated into microfluidic device with TEER measurements. (**D**) Bioreactors with small chambers are integrated with TEER sensors.

Integrating sensors into organ-on-a-chip devices is advantageous for real-time monitoring of cellular activity on a chip. Studies have incorporated physical, electrical, electrochemical, and optical sensors into microfluidic devices [31,32]. For kidney-on-a-chip, sensor integration has primarily focused on electrical measurements (e.g., TEER) due to the ability to monitor resistance as a surrogate for epithelial barrier function. Electrical measurements of resistance or impedance are widely used to evaluate the barrier function of epithelial cell monolayers [121]. The working principle of TEER measurements and sensors integration in microfluidic devices with different designs is shown in Figure 4. The TEER sensor is either designed with electrodes perpendicular or parallel to the microchannel. EVOM2 and potentiostat are commonly used to measure the resistance across the cell monolayer. Devices with pH and oxygen sensors can provide additional information. We developed a microfluidic device with integrated TEER measurement [23]. The device comprised parallel channels with a polycarbonate membrane to separate the top and bottom chambers. TEER electrodes were used to monitor tight junction integrity and cell growth. A calcium switch was used to show that the removal of calcium resulted in a significant reduction in TEER. Ag/AgCl electrodes were embedded into a two-layered microfluidic device with co-cultured epithelial and endothelial cells for evaluating barrier function [122]. Recently, Nicolas et al. developed a microfluidic titer plate, the OrganoPlate, consisting of a microtiter plate with 40 microfluidic chips [123]. An Organo TEER device with stainless-steel electrodes was designed to measure the impedance between the apical and basal sides of the tube to facilitate four-terminal sensing. Caco-2 and RPTEC cells were plated in the microchip. FITC and TRITC dextran were added to the apical channel to evaluate barrier permeability. The results showed an increase in TEER over time and a decrease in TEER when the cells were exposed to toxic compounds.

Additional functionalities are beginning to be integrated into kidney-on-a-chip devices. Asif et al. designed a platform for the real-time monitoring of TEER and pH values of media in a proximal tubule microfluidic model [124]. The transparent electrodes were created by an indium tin oxide (ITO) screening–printing technique. A portable microscope was developed for real-time monitoring of cell growth. Kidney epithelial cells (HK-2) and fibroblasts were mixed and plated in the microchannel with 5 dyn/cm^2^ FSS. An optical pH sensor was connected to the outflow of the device. After 5 days, high glucose media were used to create a pro-inflammatory environment. Then, metformin-containing media were circulated to mitigate the pro-inflammatory response. The device detected the TEER and pH value changes when the cells were cultured with high glucose media or metformin. Cohen et al. developed a kidney-on-a-chip by combining vascularized proximal tubule spheroid with tissue-embedded sensors for investigating drug-induced nephrotoxicity [125]. The device was fabricated by laser-cutting nine microwell bioreactors and embedded with oxygen sensors. Rat microvascular endothelial cells with human primary proximal tubule cells (hPTCs) were plated in microwells. The cells were exposed to 0.75 dyn/cm^2^ FSS. The bioreactor outflow was connected to a biosensor array containing electrochemical sensors, and an on-chip potentiostat (PSTAT). The electrochemical sensor arrays detected glutamine, glutamate, glucose, and lactate. The sensor-integrated microfluidic device captured the loss of polarization leading to glucose accumulation and subsequent lipid buildup and toxicity. Such multifunctional devices have the potential to elucidate disease mechanisms that may guide therapeutic responses.

## 6. Conclusions, Challenges, and Future Perspectives

Kidney-on-a-chip models have wide-ranging applications for studying fundamental renal physiology, drug screening, disease modeling, and tissue engineering. It has become increasingly clear that incorporating mechanical signals into organs-on-a-chip is important in many organs and tissues, including the kidney [35]. These stimuli can be passive and include modulating substrate stiffness, composition, topography, or geometry. Additionally, the application of externally applied forces such as FSS has wide-ranging effects on cell differentiation and function. Pathological mechanical signals can also be incorporated into in vitro devices to model acute or chronic kidney diseases. Kidney injury can alter transient fluid flows and shear stresses in the glomerulus or the tubule, and chronic injury can alter ECM mechanics or composition. Modeling these effects in microfluidic and 3D cell culture models may provide insight into normal kidney physiology and guide therapeutic approaches to mitigate kidney damage in response to acute or chronic damage.

One of the challenges of recapitulating kidney function on a chip is the complexity of renal tissue and the large number of interconnected cell processes that regulate overall tissue function. Most kidney-on-a-chip devices focus on a single tissue compartment such as the glomerulus or the proximal tubule. However, complex interactions between tissue compartments ultimately control organ-level function. For example, interactions between the tubule and the glomerulus are important for regulating filtration rate based on tubular salt concentrations through reciprocal signaling between the glomerulus and the tubule [126]. As another example, complex interactions between the tubular epithelium and the interstitial compartment are critical in the response to acute and chronic kidney injury. Crosstalk between different cell populations in the kidney is important in regulating normal kidney physiology and in the pathogenesis and progression of disease [127]. These processes are difficult to model in vitro, and to date, relatively little effort has focused on integrating multiple tissue compartments in kidney-on-a-chip. As fabrication techniques such as 3D printing or other prototyping technologies continue to be improved, additional complexity is likely to be integrated into kidney chips to better model overall tissue function.

With regard to modeling the biophysical factors that regulate kidney function, better characterization of the in vivo microenvironment is likely to improve our ability to replicate these parameters in vitro. Kidney tubular fluid shear stresses are relatively well defined based on previous micropuncture studies of kidney flow rates and the more recent application of intravital imaging for the real-time monitoring of kidney function in live animals [128,129]. The mechanical microenvironment in the glomerulus is quite complex, with glomerular endothelial cells and podocytes being subject to complex flow regimes due to fluid dynamics across the capillary wall. Modeling and simulation efforts are beginning to elucidate these mechanical parameters. Experimental measurements to validate the analytical or simulation approaches are needed to better define fluid dynamics and tissue stresses in the glomerulus and the tubule. Additional efforts to define tissue and matrix mechanics in health and disease will also likely aid in developing more physiologically relevant kidney-on-a-chip models. Most current systems rely on synthetic materials such as PDMS or other polymers to construct devices. The mechanical properties of these materials do not reflect the in vivo tissue mechanics. As tissue and ECM mechanics become better defined, particularly in disease, efforts to mimic ECM or substrate stiffness in kidney chips may improve their overall function with regard to maintaining the differentiated phenotype of a particular cell type or for capturing important physiological parameters such as the contribution of the GBM to glomerular filtration.

Finally, there is a paucity of active instrumentation and sensing mechanisms incorporated into kidney chips for real-time readouts of functional parameters. One exception is the integration of TEER as a surrogate for epithelial permeability. The ability to integrate sensors onto kidney chips to evaluate tubular epithelial drug toxicity in real time, monitor the loss of glomerular permselectivity through fluorescence analysis or albumin biosensing, or measure specific analytes in the filtrate such as creatinine or urea could add significant additional functionality to kidney-on-a-chip. This sensing technology could also provide a real-time measure of how mechanical factors applied within devices alter important cell behavior such as barrier function or monitoring cell viability. Other organ-on-a-chip systems have begun to incorporate such sensing mechanisms, and these approaches are likely to carry over to kidney-on-a-chip.

## Data Availability

Not applicable.
